# Intussusception of a Meckel’s Diverticulum in a 58-Year-Old Female: A Case Report

**DOI:** 10.7759/cureus.109567

**Published:** 2026-05-24

**Authors:** Martín Rodríguez-Garza, Edgar A Rodriguez, Sergio E Nava, Hugo Enrique Hernandez Gamboa

**Affiliations:** 1 General Surgery, Institute for Social Security and Services for State Workers (ISSSTE) Hospital Regional de Monterrey, Monterrey, MEX

**Keywords:** acute abdomen, case report, diverticulectomy, intestinal obstruction, meckel's diverticulum

## Abstract

Meckel's diverticulum is the most common congenital anomaly of the gastrointestinal tract. Although it is often asymptomatic in adults, it can present with severe complications such as diverticulitis, hemorrhage, or intestinal obstruction. Due to its clinical presentation, it frequently mimics acute appendicitis, posing a significant diagnostic challenge.

A 58-year-old female with no relevant surgical history presented to the emergency department with a two-day history of progressive sharp pain in the right iliac fossa, fever, and positive peritoneal signs. Computed tomography suggested complicated acute appendicitis. However, emergency exploratory laparotomy revealed a gangrenous Meckel’s diverticulum located 70 cm from the ileocecal valve, and intraoperatively, an intussusception of the diverticulum was identified, causing secondary intestinal obstruction. Wedge resection was executed, given the small scale of the lesion, and to minimize bowel trauma, thereby reducing the extent of the incision and requiring fewer suture stitches, along with intestinal decompression. The patient had an uneventful recovery and was discharged on the fourth postoperative day.

Meckel’s diverticulitis in the adult population is rare and often represents an intraoperative challenge. While CT scans are valuable for ruling out other pathologies, they may not always provide a definitive preoperative diagnosis. An intussusception of the diverticulum leads to bowel obstruction secondary to impaired peristalsis, a condition exacerbated by the increase in peristaltic waves. Surgical intervention remains the definitive treatment, with options ranging from simple diverticulectomy to segmental bowel resection depending on the diverticulum's characteristics and the state of the adjacent tissue.

Although more prevalent in children, Meckel’s diverticulum must be included in the differential diagnosis of adult patients presenting with symptoms of acute abdomen or intestinal obstruction. Early surgical exploration is critical to achieving a definitive diagnosis and preventing further morbidity. Intussusception is an extremely rare finding in a Meckel's diverticulum; it is important to know this case presentation, the pathophysiology, and diagnosis, so the surgical management can be effective for the patient's benefit.

## Introduction

A Meckel's diverticulum (MD) is a congenital anomaly of the small intestine wall, resulting from the incomplete atrophy of the vitelline duct in the embryo; as a result, an outpouching forms on the antimesenteric border of the small intestine wall made up of mucosa, muscular layer, and serosa, which is considered a true diverticulum because it contains all the layers of the small intestine [1,2]. The name derives from the German anatomist Johann Friedrich Meckel, who described this entity at the beginning of the 19th century.

MD is a subtle feature of human anatomy that is sometimes recognized as an incidental finding during abdominal surgery for another cause. It is the most common congenital anomaly of the gastrointestinal tract, found in 1-2% of the general population [1]. The pathophysiology of this entity is due to acute inflammation resulting from the obstruction of the diverticular lumen, whether by an enterolith, inflammatory tissue, food, foreign bodies, or tumor [2,3].

Men and women are equally affected. However, symptomatic cases occur more frequently in men than in women, in a ratio of 2-4:1. Among individuals with MD, approximately 5-6% will develop symptoms. Therefore, it is most frequently discovered incidentally, and there is no consensus on its management. Symptomatic diverticula usually present before two years of age. It is frequently described by the “rule of 2s”: 2% of the population, 2 ft from the ileocecal valve, 2 in long, two types of heterotopic mucosa (gastric and pancreatic), and presentation before two years of age [4].

The risk factors that increase the probability of the disease becoming symptomatic are the following: age under 50 years, male gender, diverticulum length greater than 2 cm, and the presence of heterotopic tissue (gastric, pancreatic, colonic). The presence of at least two of these factors increases the probability of developing symptoms by 25% [5].

Uncomplicated or complicated diverticulitis can simulate acute appendicitis, which is the most important differential diagnosis and represents a diagnostic challenge [6].

Obstruction is the presenting syndrome in 14 to 40% of symptomatic diverticula in adults and can have several causes, but the dominant cause is intussusception [7].

The diagnostic approach for this pathology is very broad; from laboratory studies, which show findings such as normochromic or hypochromic microcytic anemia due to blood loss, leukocytosis, and elevation of acute-phase reactants in cases of diverticular inflammation, but they remain nonspecific tools for diagnosing the disease since they can be present in other inflammatory/infectious diseases [8].

Contrast-enhanced abdominopelvic computed tomography (CT) provides an adequate evaluation. CT can diagnose MD as a cause of intestinal obstruction, inflammation, or perforation, rule out alternative diagnoses such as appendicitis, and guide surgical treatment [1-3]. However, not all techniques are available in different hospital centers.

When the suspicion is high, even if imaging studies are inconclusive, the definitive diagnosis is made through surgical exploration; therefore, surgical treatment should not be delayed when there is a high suspicion. In fact, exploratory laparoscopy is the investigation that most often leads to the correct diagnosis [2].

The therapeutic management of MD is defined according to the clinical context in which it is found. In the event that it is an incidental finding during abdominal surgical exploration for another cause, the treatment is selective and is based on the clinical status.

In the symptomatic clinical context of MD, the treatment is surgical, through diverticulectomy or intestinal resection and primary anastomosis, with postoperative results being comparable for both techniques.

In patients over 50 years of age, regardless of gender, surgical resection of the diverticulum is not recommended if it is an incidental intraoperative finding because the complication rate in this age group is low and decreases as age advances. Surgical resection will be performed in this age group only if it is greater than 2 cm in size or has a palpable abnormality, such as ectopic tissue that can bleed, ulcers, fibrous tissue, or tissue suspicious for malignancy [1,2,5,6].

## Case presentation

A 58-year-old female with no significant pathological or surgical history arrived to the emergency department of our hospital with the following symptoms: sharp abdominal pain localized in the right iliac fossa, positive rebound tenderness sign, psoas maneuver, positive Rovsing's and Blumberg's signs, abdominal distension with decreased peristalsis, nausea without vomiting, and low-grade fever; all of the above with two days of evolution and progressive worsening.

Preoperative laboratory findings

Upon hospital admission, the patient's laboratory findings were as follows: white blood cell (WBC) count 12.19 (elevated), absolute neutrophils 8.55 (elevated), hemoglobin 15.10, hematocrit 44.80, and alkaline phosphatase (ALP) 100.

Imaging studies

It was decided to perform a CT, which presents the following report from the radiology department:

In the present study, small intestine loops of increased caliber and air-fluid levels and normal density are observed (Figure 1 and Figure 2). The colonic frame shows the presence of fecal matter and intestinal gas. In the right iliac fossa, in the topography of the appendix, a loop with increased thickness of up to 21 mm in diameter and a 5 mm wall is observed (Figure 1), along with an increase in the density of the periappendiceal fat and scarce free fluid and free air (Figure 2). Other solid organs were unremarkable. The images are suggestive of complicated appendicitis, while not excluding mechanical obstruction secondary to a concomitant inflammatory process of alternate etiology.

**Figure 1:**
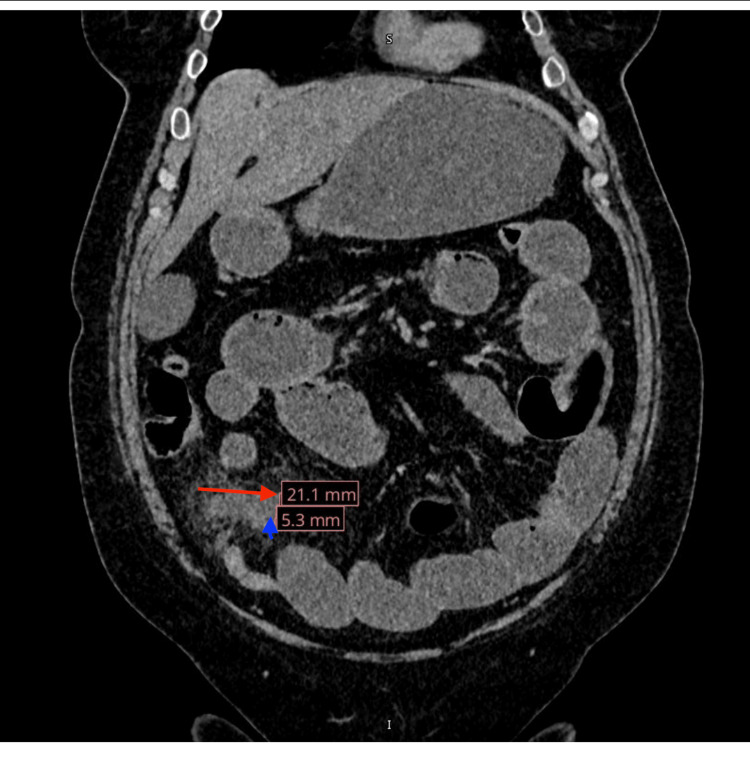
Coronal Abdominal CT Scan A loop with increased thickness of up to 21 mm in diameter (red arrow) with a 5 mm wall thickness (blue arrow).

**Figure 2:**
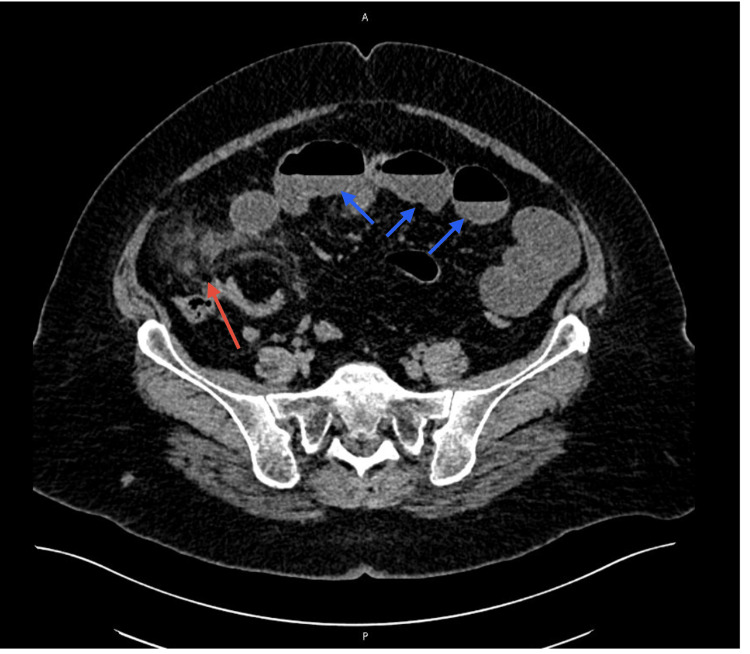
Axial CT Scan of the Abdomen The red arrow indicates the area of fat stranding and inflammation. The blue arrows indicate the dilation of bowel loops as well as the presence of air-fluid levels. CT signs of intussusception, like the target sign, were completely absent.

Therapeutic intervention 

Therapy with antibiotics and analgesics is initiated, the current state of the patient and the reserve of packed red blood cells are reviewed, and it is decided to perform emergency surgical exploration. Under balanced general anesthesia, a supraumbilical and infraumbilical incision is made, dissection is performed in layers to reach the abdominal cavity, exploration is carried out from the ligament of Treitz to the ileocecal valve, finding a gangrenous MD 70 cm from the valve, with an adhered segment of the ileum finding the site of occlusion (Figure 3), manual reduction of the intussusception was attempted without success, proximal and distal Babcock forceps are placed, a wedge resection of the diverticulum is performed, a Poole cannula is introduced through the ileum and intestinal decompression is performed, proceeding to close the ileum in two layers with 2.0 Vicryl and 3-0 vascular Prolene, adequate passage of intestinal content through the primary closure without leaks is corroborated, a Penrose-type drain is placed toward the pelvic cavity, cavity washing is performed.

**Figure 3:**
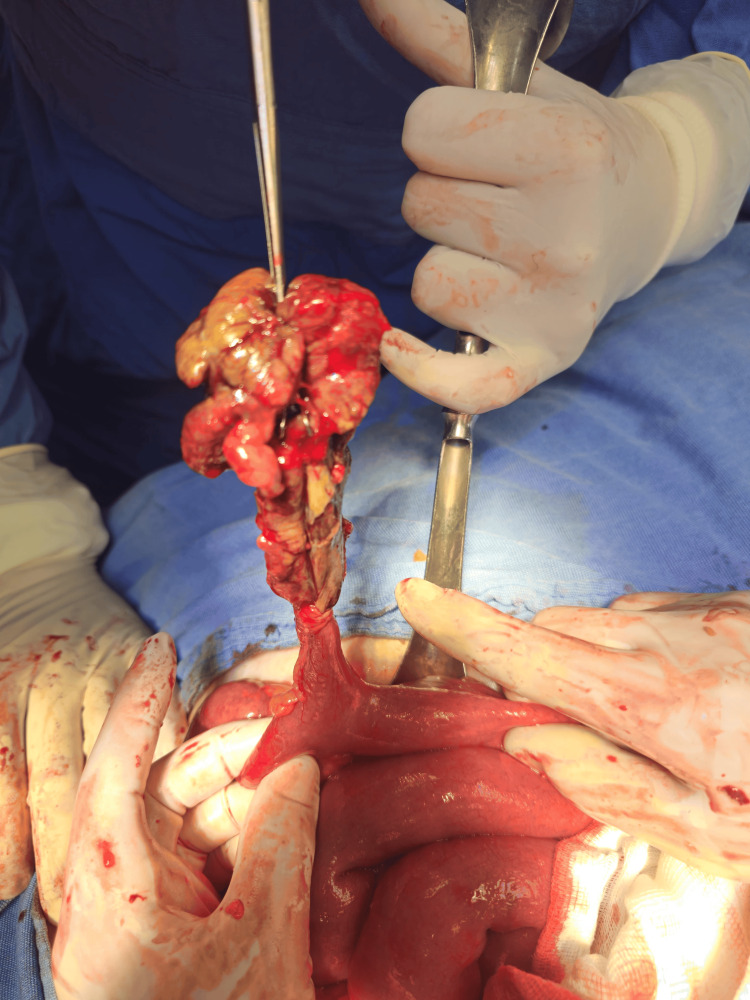
Identification of Diverticulum Note the gangrenous diverticulum with an area of intussusception at its base.

Pathology microscopic description

Histological examination of the diverticulum sections reveals a true diverticulum wall containing all normal architectural layers of the small intestine (mucosa, submucosa, muscularis propria, and serosa).

The mucosa shows widespread, transmural coagulative necrosis, extensive ulceration, and sloughing of the epithelium. The submucosa and muscularis propria exhibit severe congestion, marked edema, and extensive interstitial hemorrhage, accompanied by a dense, diffuse polymorphonuclear (neutrophilic) inflammatory infiltrate. Thrombi are noted within several small mesenteric and intramural blood vessels.

The serosal layer is covered by a dense acute inflammatory exudate composed of fibrin and neutrophils. The surgical margins show acute inflammation and vascular congestion but remain free of frank necrosis.

Postoperative course

The patient remained hospitalized for four days. She started an oral liquid diet on the second postoperative day, tolerating it adequately, and with progression of the diet, with the presence of flatus and bowel movements. Penrose drain was found with serohematic discharge and minimal output; the patient was discharged from our hospital and followed up in the outpatient clinic.

## Discussion

This case shows an atypical presentation of Meckel’s diverticulitis with intussusception in a 58-year-old woman, which is, by epidemiology and statistics, less than 2% of all MD cases. Differentiating between acute appendicitis, colonic diverticulitis, Crohn’s disease, and other causes of acute abdominal pain is the first challenge for a surgeon to find the correct answer. Here, we see the relevance of radiological images (which still provided an inconclusive diagnosis) and the importance of surgical intervention, which revealed the final cause of the patient’s symptoms. The importance of this case report lies in the lack of cases in this patient context (age, clinical presentation, inconclusive image report, intestinal occlusion, intussusception as a surgical finding) and how we can manage a patient with these characteristics.

MD can cause intestinal obstruction, which presents with abdominal pain, nausea, vomiting, and distension. Intestinal obstruction can be secondary to (1) volvulus around the vitelloumbilical cord, (2) intussusception, (3) inflammation with adhesion, (4) a band between the MD and mesenterium, and (5) internal hernia or Littre hernia [7,8].

Intussusception caused by MD is estimated to account for 4% of all intestinal obstruction cases related to intussusception [7]. Preoperative diagnosis can be difficult because, clinically, patients with intussusception frequently exhibit symptoms that overlap with those of other conditions, such as other causes of acute abdomen [7].

The question then was to determine what was the best management and treatment of MD once diagnosed. In most literature, the accepted treatment modality for an MD is diverticulectomy or segmental resection, as the risk of recurrence remains significant after reduction alone [9]. We chose to perform a diverticulectomy for these reasons: Our patient had a focal area of necrosis on the top of the MD without damage to the base of the diverticulum. The base of the diverticulum was thick enough so we could perform a primary closure of the surgical wound without compromising the patency of the intestinal lumen. There are mixed reviews on the standard of treatment for MDs; however, both diverticulectomy and segmental resection appear to be safe treatment modalities with high patient tolerance [1,2,9]. 

Surgical specialists must remain cognizant of the most prevalent congenital anomaly of the small intestine. As demonstrated by the clinical scenario described herein, a thorough understanding of such presentations underscores the critical importance of timely surgical exploration and prompt definitive management to mitigate adverse outcomes.

## Conclusions

MD is a frequent entity in children under two years of age. It represents 1-2% in adults incidence of acute abdomen cases. Just as in our case, it represents a diagnostic challenge because it is not the first diagnostic possibility in adults with abdominal pain, with entities such as acute appendicitis, diverticulitis, inflammatory bowel disease, or benign or malignant tumors of the small intestine being more frequent. If there is high suspicion, surgical treatment should not be delayed because it is considered diagnostic/therapeutic. Among surgical procedures, the accepted treatment modality for an MD is diverticulectomy or segmental resection, as the risk of recurrence remains significant after reduction of intussusception alone. In our case, a diverticulectomy via wedge resection was executed, given that the MD exhibited a narrow base, and neither the gangrenous nor the intussuscepted tissue extended to this margin, so we could perform a primary closure of the surgical wound without compromising the patency of the intestinal lumen. Although the patient experienced an uneventful postoperative course with no evidence of bowel obstruction during the initial post-surgical weeks, the brief duration of the follow-up period precludes the exclusion of potential long-term complications, representing a recognized limitation of this case report.
